# Research on Threshold Optimization and Variability-Based Digital Biomarker Approaches Through MMSE-Lifelog Multimodal Integrated Analysis from a Clinical Screening Perspective

**DOI:** 10.3390/healthcare14081094

**Published:** 2026-04-20

**Authors:** Yeeun Park, Jin-hyoung Jeong

**Affiliations:** 1 Department of Electronic and Communication Engineering, Catholic Kwandong University, Gangneung-si 25601, Republic of Korea; 565dpdms@cku.ac.kr; 2 Department of Biomedical Management, College of Medical Convergence, Catholic Kwandong University, Gangneung-si 25601, Republic of Korea

**Keywords:** mild cognitive impairment, digital biomarker, threshold optimization

## Abstract

**Background**: Early screening of cognitive impairment is essential for timely clinical intervention; however, conventional cognitive tests such as the Mini-Mental State Examination (MMSE) rely on fixed thresholds that may not be optimal in real-world screening settings. **Methods**: This study developed a threshold-aware multimodal screening framework integrating MMSE item-level scores with wearable-derived sleep and physical activity lifelog data. A dataset of 174 adults was analyzed and categorized into cognitively normal (CN), mild cognitive impairment (MCI), and dementia, with MCI and dementia combined as an impaired group. A CatBoost-based binary classification model was trained using five-fold cross-validation. The optimal decision threshold was determined by maximizing balanced accuracy using out-of-fold predictions. **Results**: The optimized threshold (0.49) achieved an accuracy of 0.818 and a balanced accuracy of 0.728 on the validation set. The recall values were 0.885 for CN and 0.571 for the impaired group, with an area under the ROC curve of 0.676. Feature importance and stability analyses showed that variability-related sleep and activity features were consistently selected across folds. **Conclusions**: These findings suggest that threshold optimization combined with multimodal lifelog integration may improve the interpretability of screening decisions. Variability-based lifelog features may provide complementary information alongside MMSE, although their role remains exploratory and requires further validation in larger and longitudinal cohorts.

## 1. Introduction

Mild cognitive impairment (MCI) is defined as a transitional stage between normal aging and dementia, characterized by measurable declines in cognitive functions, including memory, while daily functional abilities remain relatively preserved [1]. Individuals with MCI are reported to have an annual risk of progressing to Alzheimer’s disease or other forms of dementia at a rate of approximately 10–15%, highlighting the importance of early detection and timely intervention [2]. To address this clinical need, brief cognitive screening instruments (CSIs), such as the Mini-Mental State Examination (MMSE) and the Montreal Cognitive Assessment (MoCA), have been widely used in both clinical and research settings to identify individuals at risk of cognitive decline [3,4]. Among these instruments, the MMSE has been used for several decades as a standard cognitive screening tool worldwide because it enables rapid assessment of multiple cognitive domains, including orientation, memory, attention, language function, and visuospatial abilities [5,6].

Despite its widespread clinical use, accumulating evidence suggests that MMSE-based screening has substantial limitations. Several studies have reported variability in the diagnostic performance of the MMSE when used to predict conversion from MCI to dementia [7,8]. A Cochrane systematic review evaluating the ability of the MMSE to predict dementia conversion in individuals with MCI reported sensitivity values ranging from 23% to 76% and specificity values ranging from 40% to 94%, demonstrating considerable heterogeneity across studies [9]. These findings suggest that the use of the MMSE alone at a single time point provides insufficient evidence for predicting future dementia progression. One major contributor to this variability is the heterogeneity in cut-off score determination. MMSE scores are strongly influenced by demographic factors such as age and educational level, and applying a uniform cut-off score across different populations can lead to substantial differences in false negative and false positive rates [10,11]. Although the MoCA was developed to improve sensitivity for detecting MCI and to address some limitations of the MMSE [12], subsequent studies have shown that its optimal cutoff point also varies significantly depending on population characteristics and research settings [13,14]. For example, a meta-analysis reported that the originally recommended MoCA cutoff may substantially increase false positive rates, while lower cutoff values may provide better diagnostic performance in certain populations [15]. Furthermore, several systematic reviews have demonstrated that optimal thresholds for both the MMSE and MoCA vary according to cultural background, educational level, and study design [16,17,18]. In addition, the method used to determine cutoff points can influence diagnostic performance [19]. For instance, thresholds derived using the maximum Youden index may produce different optimal operating points compared with those derived using overall accuracy, thereby altering the trade-off between sensitivity and specificity and potentially affecting misclassification rates in real-world screening environments [20,21]. Collectively, these findings highlight several inherent limitations of traditional cognitive screening tests, including limited predictive power due to single-time-point assessments, demographic biases, and heterogeneity in threshold determination strategies [22,23].

To address these limitations, recent research has increasingly explored the use of digital technologies for the continuous and ecologically valid monitoring of cognitive health. Studies using wearable sensors, mobile devices, and smart home systems have demonstrated that behavioral and physiological signals collected in real-world environments can provide valuable information for detecting early cognitive decline [24,25]. In particular, lifelog data that are passively collected during daily life, such as physical activity levels, sleep patterns, mobility behavior, and computer usage patterns, have emerged as promising digital biomarkers that may reflect changes in cognitive status. For example, research using wrist-worn sensors has reported that poor sleep quality is associated with memory deficits in individuals with amnestic mild cognitive impairment (aMCI) [26]. Similarly, GPS-based analyses of mobility patterns have shown potential in distinguishing cognitively normal individuals from patients with MCI and Alzheimer’s disease [27]. Subsequent systematic reviews have further suggested that wearable and embedded sensor technologies can objectively capture behavioral changes associated with cognitive and functional decline. These studies consistently report patterns such as reduced daily activity levels, decreased sleep efficiency, and increased fragmentation of circadian rhythms in individuals with dementia. Such findings indicate that lifelog-based data may serve as valuable complementary information to traditional one-time cognitive assessments. In addition, aging-related comorbidities such as hypertension, diabetes, and metabolic instability have been reported to influence the progression and clinical manifestation of neurodegenerative disorders. These factors may contribute to heterogeneity in cognitive trajectories, further emphasizing the need for context-aware and adaptive screening strategies that go beyond single-time-point cognitive assessments [28].

Nevertheless, digital biomarker research still faces several important challenges. Some reviews have pointed out that digital biomarker technologies are not yet sufficiently standardized and that there is limited consensus regarding which behavioral features are the most reliable indicators of cognitive decline. In addition, approaches relying solely on lifelog data may suffer from small sample sizes, substantial heterogeneity in individual behavioral patterns, and limited generalizability across external cohorts. Consequently, integrating lifelog-derived behavioral signals with established clinical cognitive tests remains an important research challenge.

To overcome these limitations, recent studies have begun to integrate traditional cognitive assessment scores with digital biomarkers using machine learning-based multimodal models [29]. This approach aims to simultaneously leverage the diagnostic reliability of established clinical tests and the ecological validity of real-world behavioral data. For example, multimodal models that combine biosignals with digital cognitive tests have been reported to achieve higher classification accuracy than single-modality models [30]. Similarly, machine learning models integrating gait characteristics, body composition measures, and sleep parameters have demonstrated promising diagnostic performance in classifying MCI severity. Despite these advances, several limitations remain. A systematic review of digital cognitive biomarker research reported that although such approaches may achieve diagnostic performance comparable to traditional cognitive tests, the evidence supporting their independent use in clinical practice remains limited [31]. Furthermore, a recent scoping review analyzing artificial intelligence-based MCI detection models reported an average area under the curve (AUC) of approximately 0.821, but only a small number of studies conducted external validation or probability calibration [32]. Another important limitation concerns the choice of model evaluation metrics. Many multimodal studies primarily evaluate model performance using summary metrics such as overall accuracy or AUC. However, in real-world clinical screening contexts, false negatives—cases in which cognitively impaired individuals are incorrectly classified as normal—may have far more serious consequences than false positives. Previous research has shown that optimal thresholds derived using the maximum Youden index or overall accuracy may produce different operating points, which can substantially influence screening performance [33].

Taken together, these findings indicate several important research gaps. First, although multimodal models integrating traditional cognitive test scores with digital biomarkers are increasingly being investigated, relatively few studies have systematically optimized decision thresholds specifically for clinical screening purposes [34,35]. Second, many existing studies focus primarily on summary performance metrics such as overall accuracy or AUC, with limited analysis of how the trade-off between sensitivity and specificity varies across different operating points [36,37]. Third, while temporal variability in behavioral patterns—rather than average levels of lifelog signals—has been suggested as a potential indicator of cognitive decline [38], it remains unclear whether such features can be reliably selected as candidate digital biomarkers within multimodal models using cross-validation-based feature stability analysis.

Therefore, in this study, we developed a multimodal machine learning framework that integrates MMSE item-level scores with wearable-derived sleep and physical activity lifelog data. Using out-of-fold (OOF) predictions obtained from five-fold cross-validation, we explored decision thresholds that maximize balanced accuracy in order to identify operating points that minimize false negatives in clinical screening settings.

In addition, we evaluated whether variability-based features derived from lifelog data were consistently selected across cross-validation folds through feature stability analysis, thereby examining their potential utility as digital biomarkers reflecting cognitive decline. Finally, we conducted ablation analyses comparing MMSE-only models, lifelog-only models, and various multimodal integration models to assess how the selective incorporation of lifelog data can enhance MMSE-based cognitive screening models.

## 2. Materials and Methods

### 2.1. Dataset Composition

This study aims to detect early cognitive anomalies from a clinical screening perspective by integrating wearable-based lifelog data provided by AI-Hub with traditional cognitive test scores. The study cohort comprised 174 adults; for each subject, cognitive function test results along with sleep and physical activity lifelog data were obtained. Based on clinical diagnosis, participants were classified into three groups: Cognitively Normal (CN, n = 111), Mild Cognitive Impairment (MCI, n = 51), and Dementia (n = 12). For model training and performance evaluation, the entire dataset was stratified into a training set (n = 141) and a validation set (n = 33), ensuring that the proportions of diagnostic groups were maintained across all splits.

The lifelog data were provided as repeated time-series measurements per individual. In this study, these were aggregated into subject-level summary features and then merged with MMSE data using a unique identifier (email_key), thereby standardizing the unit of analysis to one record per subject.

To align with clinical screening objectives, a multi-stage classification strategy was adopted. Stage 1 involved a binary classification between the normal group (CN) and the cognitively impaired group (Impaired; combining MCI and Dementia). The primary goal of this stage was to minimize the number of impaired patients misclassified as normal in a clinical setting. Stage 2 involved a subsequent analysis within the impaired group to distinguish between MCI and Dementia; however, interpretations of these results warrant caution due to the limited sample size of the dementia group.

### 2.2. Traditional Cognitive Test Data (MMSE)

Cognitive assessment was based on the Korean version of the Mini-Mental State Examination (MMSE). Both the total score and item-level scores (Q01–Q19) were included in the analysis. The MMSE data consisted of clinical cognitive screening results administered at a single time point by trained evaluators, constituting one record per subject. By incorporating item-level scores alongside the total score, this study sought to reflect not only the overall cognitive level but also specific performance characteristics across different cognitive domains as model input variables. Items Q01–Q19 evaluate distinct cognitive areas such as orientation, memory, attention, language function, and visuospatial construction.

MMSE-related data were used as model input variables in their raw form without additional aggregation or transformation, while the clinical diagnosis (DIAG_NM) served as the dependent variable for the classification model.

Since the source MMSE data from AI-Hub contained some items with sub-components (Q11, Q12, Q13, Q14, Q16), the total score was recalculated to accurately reflect the item structure. Furthermore, item scores were converted to a binary (0/1) coding system before calculating the total score. The recalculated total scores showed perfect consistency with the original TOTAL variable across all training and validation samples (mismatch = 0).

### 2.3. Lifelog Data Preprocessing and Feature Extraction

Lifelog data collected via wearable devices comprised two modalities: Sleep and Physical Activity (Walk/Activity). These data were based on continuous monitoring within the participants’ real-world daily living environments. The lifelog data consisted of time-series measurements repeated on a daily or session basis, containing thousands of observations per individual. In this study, these repeated measurements were aggregated to generate features for subject-level analysis.

For both sleep and activity data, summary statistics including mean, standard deviation (SD), minimum, and maximum were calculated for each individual. Notably, by including the standard deviation and range indicators alongside simple averages, the study aimed to capture the variability of behavioral patterns. This approach aligns with recent digital biomarker research, which has repeatedly reported associations between behavioral variability and cognitive decline [39].

To address missingness in the raw lifelog streams, subject-level summary statistics were calculated using all available observations for each individual within each modality. No additional imputation, interpolation, or model-based reconstruction of missing time points was applied. This approach was adopted to preserve observed behavioral patterns while reducing sensitivity to irregular recording density across participants. The aggregated lifelog features were merged with the MMSE data based on the unique identifier (email_key). Consequently, datasets were constructed for single modalities (MMSE only, Sleep only, Activity only), bi-modal combinations (MMSE + Sleep, MMSE + Activity), and a multimodal combination integrating all three modalities.

### 2.4. Classification Model and Training Strategy

This study utilized the CatBoost gradient boosting algorithm for classification. CatBoost is a tree-based ensemble model known for its stable performance in environments with small sample sizes and high-dimensional data, as well as its effectiveness in learning non-linear interactions between numerical variables [40]. Given that the study data had a limited sample size but a relatively large number of variables due to lifelog aggregation—along with potential non-linear interactions—tree-based models like CatBoost were deemed more suitable than deep learning models (which carry a high risk of overfitting) or logistic regression (which relies on linear assumptions). Specifically, CatBoost was selected as the final model because the study prioritized model stability in cross-validation and the interpretability of feature importance over simple performance maximization, which are critical requirements in clinical screening research. Although more complex deep learning architectures, such as recurrent neural networks or Transformer-based models, may be useful for modeling raw longitudinal sensor streams, the present study used subject-level aggregated statistical features rather than raw temporal sequences. Under these conditions, and given the relatively limited sample size, a tree-based ensemble model was considered more appropriate to ensure model stability, interpretability, and reduced risk of overfitting.

Additionally, CatBoost supports training without separate normalization of input variables, making it advantageous for integrating lifelog metrics with varying units and distributions. Furthermore, it allows for the analysis of feature importance to interpret the relative contribution of each feature, enabling the presentation of results in a clinically explainable manner [41,42].

Model training was performed using 5-fold cross-validation, maintaining the same stratified data splitting strategy in each fold. In each fold, the model was trained on four folds and evaluated on the remaining fold to generate out-of-fold (OOF) predictions. Performance evaluation focused on balanced accuracy, a metric suitable for clinical screening, rather than simple accuracy.

All experiments were conducted in a CPU-only environment on Windows 10 (build 19045) using Python (v3.12.7), scikit-learn (v1.5.1), CatBoost (v1.2.8), NumPy (v1.26.4), pandas (v2.2.2), and PyTorch (v2.9.1).

The multimodal input feature vector for each subject was constructed by concatenating features from multiple modalities. Let X^(MMSE)^, X^(sleep)^, X^(activity)^ denote the feature vectors derived from the MMSE item scores, sleep lifelog data, and activity lifelog data, respectively. The overall multimodal feature vector XXX for each subject can be expressed as

X = [X^(MMSE)^, X^(sleep)^, X^(activity)^]

where the dimensions of the feature vectors correspond to the number of variables extracted from each modality.

Given the feature vector Xi for subject i, the CatBoost model learns a mapping function f(⋅) that estimates the probability of cognitive impairment as

$$\hat{p_{i}}=f(X_{i})$$

where $\hat{p_{i}}$ ∈ [0, 1] represents the predicted probability that subject iii belongs to the cognitively impaired class. The final class label ŷᵢ is determined based on a decision threshold τ applied to the predicted probability. To mitigate potential overfitting due to the relatively small sample size, model evaluation relied on stratified 5-fold cross-validation and Out-of-Fold (OOF) predictions.

### 2.5. Threshold Optimization and Clinical Screening Strategy

A key methodological feature of this study is that the decision threshold for the classification model’s output probabilities was not set to a fixed default but was optimized to establish an operating point aligned with clinical objectives. While the fixed threshold of 0.5 used in general machine learning research presupposes the maximization of overall accuracy [43], minimizing false negatives constitutes a more critical decision-making criterion in clinical screening environments such as cognitive dysfunction screening.

Accordingly, instead of maximizing total accuracy or the Youden Index, this study searched for a threshold that maximized balanced accuracy based on Out-Of-Fold (OOF) prediction results from the 5-fold cross-validation. The threshold was varied across a continuous interval from 0 to 1, and balanced accuracy, recall, and precision were calculated for each value. Formally, for a given decision threshold τ, the predicted class label ŷᵢ for subject i is defined as follows.

$${\hat{y}}_{i}=1\;\mathrm{if}\;\hat{p_{i}}\geq \tau$$

ŷ_i_ = 0

otherwise where $\hat{p_{i}}$ denotes the predicted probability from the CatBoost model.

To determine the optimal operating point for clinical screening, the threshold τ was selected to maximize balanced accuracy based on Out-of-Fold (OOF) predictions obtained from 5-fold cross-validation. Balanced accuracy is defined as

BA = (Sensitivity + Specificity)/2

The optimal threshold τ* was determined as

τ* = argmax(τ) BA(τ)

where BA(τ) denotes the balanced accuracy achieved when applying threshold τ.

As a result, it was confirmed that balanced accuracy for the Stage 1 classification (CN vs. Impaired) was maximized at a threshold of 0.49. By applying this threshold, the study successfully improved recall for the impaired group while limiting the rate of false positives (misclassification of the normal group).

This threshold-aware approach represents a strategy reflecting the clinical screening requirement to minimize false negatives, distinguishing this study from existing research by explicitly incorporating a decision criterion different from simple accuracy maximization. Although this study did not explicitly implement formal cost-sensitive learning, the threshold optimization strategy was designed to reflect the asymmetry of clinical screening decisions, where missing cognitively impaired individuals is generally more consequential than over-screening normal individuals. Thus, the proposed framework may be interpreted as an initial threshold-aware approximation of clinically cost-sensitive decision-making.

### 2.6. Performance Evaluation and Comparative Analysis

Model performance was analyzed comprehensively using multiple evaluation metrics meaningful from a clinical screening perspective, rather than relying on a single indicator. Performance was evaluated using both ROC curves and Precision-Recall (PR) curves, with the Area Under the Curve (AUC) and Average Precision (AP) reported [44]. Additionally, confusion matrices were used to quantitatively analyze the patterns of misclassification between the normal and cognitively impaired groups.

Furthermore, an ablation study was conducted to compare the performance of single-modality models (MMSE-only, Lifelog-only) against the multimodal combined model. This analysis served to reaffirm that while MMSE remains a robust cognitive screening tool, lifelog data—which shows limited performance on its own—can provide additive value and complementary information to clinical judgment when combined with MMSE.

## 3. Results

### 3.1. Subject Characteristics and Data Composition

A total of 174 subjects were included in the analysis. Based on clinical diagnostic criteria, the cohort consisted of 111 Cognitively Normal (CN) individuals, 51 with Mild Cognitive Impairment (MCI), and 12 with Dementia. For the first stage of analysis in this study, MCI and Dementia were combined into a single group defined as “Impaired,” in accordance with the objective of clinical screening.

As presented in Table 1, the distribution of MMSE total scores showed distinct differences depending on cognitive status. The CN group exhibited a relatively narrow variance with a mean MMSE total score of 27.70 ± 1.78, whereas the MCI group showed partial overlap with a mean of 25.82 ± 3.28. The Dementia group recorded the lowest mean score of 16.58 ± 8.03; the large standard deviation in this group suggests significant heterogeneity in cognitive function within the disease stage.

These results demonstrate the limitations of relying solely on single-time-point MMSE scores to clearly distinguish between normal and impaired groups, particularly confirming the notable overlap in score distributions at the MCI stage. Consequently, this study adopted an approach that combines MMSE scores with lifelog-based behavioral variability metrics to assist in screening for cognitive impairment risk.

The number of input variables per modality consisted of 34 MMSE item-based features, 108 sleep lifelog features, and 96 activity lifelog features. In the multimodal model integrating all three modalities, a total of 238 variables were utilized.

### 3.2. Stage 1 Classification Performance: CN vs. Impaired

The CatBoost-based binary classification model was evaluated using a threshold optimization strategy based on out-of-fold (OOF) cross-validation results, rather than applying a fixed default threshold of 0.5. Through this process, a clinically meaningful operating point was identified for the Stage 1 classification task. Specifically, this study searched for the threshold that maximized balanced accuracy in the 5-fold cross-validation framework, and a threshold of 0.49 was selected as the primary operating threshold for the Stage 1 classification distinguishing CN from Impaired.

**Figure 1:**
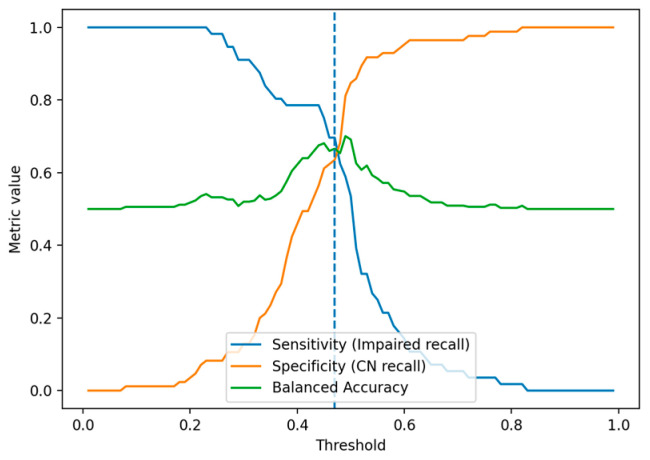
Threshold trade-off and optimized operating point.

Figure 1 visualizes the trade-off among recall (sensitivity) for the impaired group, recall (specificity) for the normal group, and balanced accuracy across varying thresholds. Balanced accuracy peaked around the 0.45–0.50 range; specifically, 0.49 was determined to be a clinically interpretable equilibrium point that constrains misclassification of the normal group without excessively sacrificing sensitivity for the impaired group.

Applying this threshold to the validation set resulted in an overall accuracy of 0.818 and a balanced accuracy of 0.728. The recall for the normal group (CN) was 0.885, and the recall for the impaired group (Impaired) was 0.571. This result corresponds to correctly screening 4 out of 7 impaired patients, indicating moderate detection performance at the screening stage even within a limited sample size environment.

The confusion matrix analysis confirmed that 23 out of 26 CN subjects were correctly classified, and 4 out of 7 Impaired subjects were correctly classified. Three cases of impaired subjects being misclassified as normal were identified.

Compared with the default threshold of 0.5, the optimized threshold was intended to reduce false negatives while maintaining relatively high specificity. However, a direct comparison between the optimized threshold (0.49) and the default threshold (0.50) on the validation set showed identical performance across all evaluation metrics, because no predicted probabilities were located between 0.49 and 0.50. Accordingly, both thresholds produced the same classification outcomes on the validation data.

To further examine the clinical adaptability of threshold selection, an additional cost-sensitive threshold analysis was conducted as a supplementary scenario-based evaluation using the validation threshold table derived from the same run. Importantly, this analysis was not intended to replace the primary OOF-derived threshold (0.49), but rather to illustrate how the preferred operating point may shift under different clinical cost assumptions.

**Figure 2:**
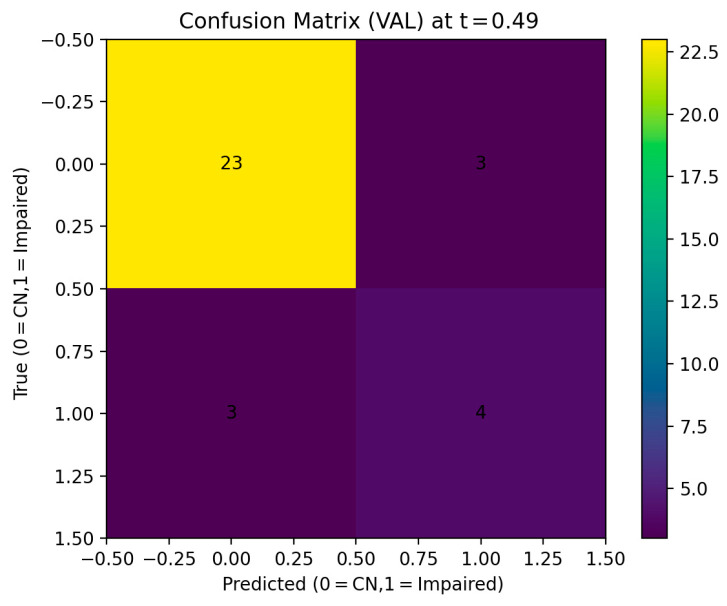
Confusion Matrix at optimized decision threshold.

Under equal-cost assumptions for false positives and false negatives, the preferred threshold was 0.54, yielding a sensitivity of 0.571, specificity of 0.885, and balanced accuracy of 0.728. When the relative cost assigned to false negatives was increased (e.g., FN cost × 3 or ×5), the preferred threshold shifted downward to 0.16, resulting in improved sensitivity (0.714) at the expense of reduced specificity (0.577). These findings suggest that while 0.49 served as the primary threshold selected from the OOF framework, the preferred operating point in practice may vary depending on the relative clinical priority assigned to minimizing missed impaired cases versus avoiding unnecessary over-screening (Table 2 and Figure 2).

### 3.3. ROC and Precision-Recall Analysis

In the ROC curve analysis, the AUC of the Stage 1 binary classification model was 0.676. Although this AUC value falls within the poor-to-fair range according to common classification benchmarks, it reflects the characteristics of a screening-oriented model designed to prioritize early detection of risk groups rather than maximizing overall classification accuracy. Notably, the True Positive Rate increased gradually in the low False Positive Rate region, indicating a characteristic capability to progressively capture the impaired group without excessively misclassifying the normal group.

In the Precision-Recall (PR) curve analysis, the Average Precision (AP) was 0.651, demonstrating that the model identifies the impaired group with a certain level of stability even in an imbalanced data environment. However, a pattern was observed where precision dropped sharply as recall increased; this is interpreted as a typical phenomenon occurring when the sample size of the impaired group is limited. This pattern highlights the inherent trade-off between sensitivity and precision in screening-oriented models, where increasing sensitivity to reduce false negatives may inevitably introduce additional false positives. This characteristic implies that pursuing higher sensitivity increases the likelihood of misclassifying some normal subjects as impaired, reflecting the trade-off inherent in risk minimization strategies during clinical screening.

**Figure 3:**
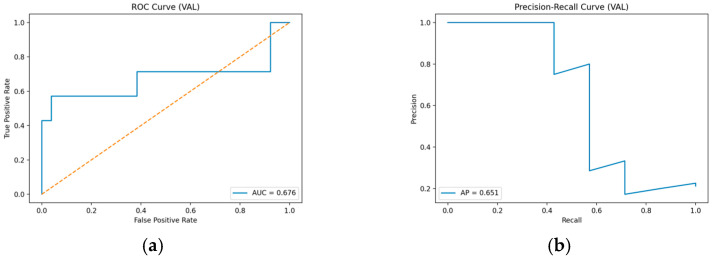
(**a**) ROC curve of the CatBoost-based CN vs. Impaired classification model; (**b**) Precision-Recall curve of the CN vs. Impaired classification model.

Therefore, the ROC and PR analysis results suggest that the model may be more appropriately interpreted as an exploratory support tool for initial screening rather than as a stand-alone diagnostic system (Figure 3).

### 3.4. Probability Calibration and Predictive Reliability Assessment

**Figure 4:**
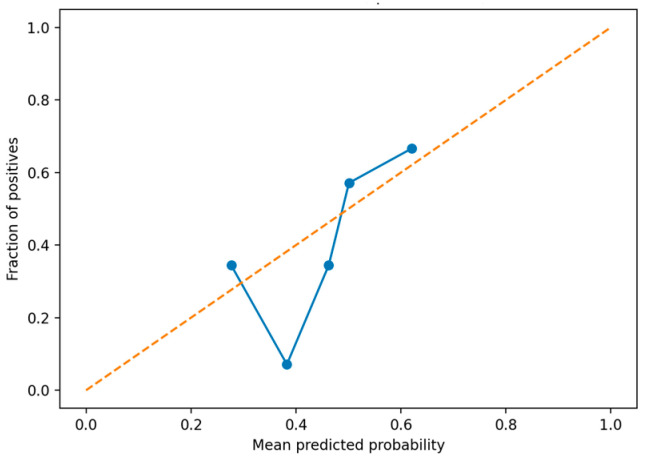
Calibration curve using quantile-based bins. The corresponding Brier score on the validation set was 0.1473.

Figure 4 presents the calibration curve of predicted probabilities on the validation set. In the mid-range probability region, the observed positive rate generally aligned with the predicted probabilities; however, under- or over-estimation was observed in some bins. This suggests that uncertainty in probability estimation may exist under limited sample size and class-imbalance conditions. To complement the visual interpretation of the calibration curve, the Brier score was additionally computed on the validation set as a quantitative calibration metric. The model yielded a Brier score of 0.1473, indicating a moderate level of probability calibration under the limited sample conditions of the present study.

Nevertheless, because the primary concern in clinical screening is not the absolute accuracy of predicted probabilities but the stability of threshold-based decision-making, the present results are considered interpretable within an acceptable range.

### 3.5. Cross-Validation–Based Threshold Stability Analysis

Across 5-fold cross-validation, fold-wise AUC values ranged from 0.628 to 0.775, with a mean AUC of 0.698 and a standard deviation of 0.061. Balanced accuracy also varied across folds, with a mean balanced accuracy of 0.692.

The optimal threshold in each fold ranged from 0.15 to 0.50, indicating that the optimal operating point can shift depending on how the data are partitioned. However, the validation-derived threshold of 0.49 fell within a range that showed stable performance across most folds, suggesting that it may represent a practically interpretable operating point under the present dataset conditions.

Despite the relatively limited dataset size, the consistency of performance metrics across cross-validation folds suggests that the model does not rely excessively on a particular data partition and maintains relatively stable decision behavior.

### 3.6. Modality-Wise Performance Comparison and Ablation Analysis

In the modality-wise comparison, the MMSE-only model achieved the highest mean AUC (0.861). In contrast, the sleep-lifelog–only model and the activity-lifelog–only model showed relatively lower performance, with mean AUCs of 0.571 and 0.612, respectively.

The strong performance of the MMSE-only model confirms the well-established diagnostic value of traditional cognitive screening tools for detecting cognitive impairment. For the multimodal models combining MMSE with lifelog data, AUC did not increase compared with the MMSE-only model, while balanced accuracy remained at a comparable level. Notably, the model combining MMSE and sleep data achieved a mean AUC of 0.769. This result suggests that sleep-related behavioral variability captured through wearable devices may provide exploratory complementary information that supports cognitive screening decisions when integrated with MMSE features. The model integrating all three modalities yielded a mean AUC of 0.698, which may reflect potential information redundancy and noise effects associated with increased feature dimensionality. In a small-sample environment, increasing the number of input variables may introduce noise and reduce generalization performance, highlighting the importance of selective modality integration rather than indiscriminately combining all available features (Table 3).

### 3.7. Feature Importance and Stability Analysis

Feature importance analysis of the CatBoost model showed that, in addition to MMSE item scores, variability-related indicators of activity and sleep patterns repeatedly appeared among the top important variables. In particular, metrics such as activity variability, dispersion within low-activity periods, and the frequency of awakenings during sleep were consistently selected as top features across multiple cross-validation folds.

**Figure 5:**
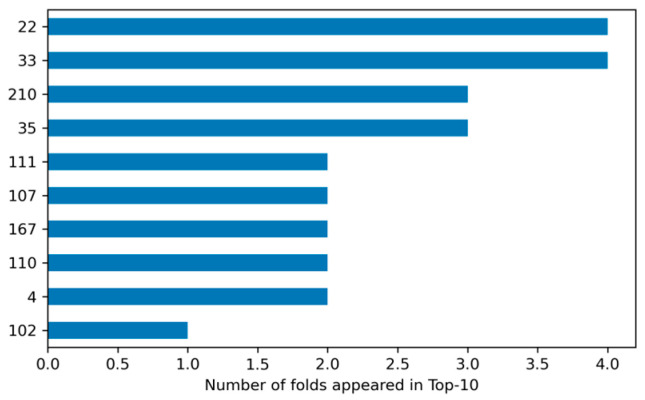
Stability of Important Features Across Cross-Validation Folds.

Feature stability analysis further indicated that several lifelog variables were included in the top 10 features in up to four folds, demonstrating consistent contributions during model training. These findings suggest that temporal variability and instability in behavioral signals, rather than absolute activity or sleep levels, may potentially reflect behavioral variability patterns associated with cognitive decline. Moreover, features repeatedly selected across multiple cross-validation folds imply that the model is not overly dependent on a particular data split and instead learns relatively consistent decision criteria. This stability supports the potential role of variability based lifelog features as exploratory digital biomarker candidates for cognitive impairment detection (Figure 5).

## 4. Discussion

This study explored a threshold-optimization strategy and the potential of digital biomarkers for an early clinical screening model to identify individuals at risk of cognitive impairment by integrating MMSE and lifelog data in a multimodal framework. Rather than focusing on maximizing classification performance alone, we adopted an approach that adjusts decision criteria from a clinical risk-management perspective and interpreted model performance primarily in terms of real-world clinical applicability.

The CatBoost-based binary classification model (CN vs. Impaired) achieved an AUC of 0.676 and an average precision (AP) of 0.651 on the validation set [45]. Although these values fall within the poor-to-fair range according to common classification benchmarks, they may still be interpretable in the context of a supportive tool aimed at detecting potential cognitive impairment at the initial screening stage. Notably, the ROC curve showed a gradual increase in the true positive rate even in the low false positive rate region, reflecting a model characteristic that can incrementally capture the impaired group while avoiding excessive misclassification of the normal group.

A key contribution of this work is that the decision threshold was not fixed at 0.5; instead, we searched for the threshold that maximized balanced accuracy based on 5-fold cross-validation results. The optimal out-of-fold (OOF) threshold was identified as 0.49. When this threshold was applied to the validation set, the model achieved an accuracy of 0.818, a balanced accuracy of 0.728, a recall of 0.885 for the normal group, and a recall of 0.571 for the impaired group. These results suggest that, even under a limited-sample setting, the model can identify a subset of impaired individuals while relatively suppressing misclassification of the normal group. In screening scenarios where false negatives are clinically important, threshold optimization may provide practical value by balancing sensitivity and specificity while helping to reduce missed at-risk cases. Additional cost-sensitive analysis further demonstrated that the preferred threshold is not fixed, but may shift according to the relative clinical importance assigned to false negatives and false positives. In particular, when false negatives were penalized more heavily, lower thresholds were favored to improve sensitivity, even at the cost of reduced specificity. This finding supports the interpretation that threshold selection in cognitive screening should be regarded as a context-dependent clinical operating parameter rather than a universally fixed cutoff. It should also be noted that the supplementary cost-sensitive threshold analysis was intended to demonstrate the context-dependent nature of threshold selection under varying clinical priorities, rather than to replace the primary OOF-derived threshold used in the main evaluation framework. However, real-world clinical screening decisions may involve heterogeneous cost structures depending on the intended use case, healthcare setting, and downstream referral burden. Therefore, future work should extend the present framework by incorporating explicit cost-sensitive learning or clinical utility-based threshold selection methods that can adapt the operating point to different screening priorities.

In addition, by employing 5-fold cross-validation with an out-of-fold prediction framework, we were able to assess the stability of model performance and threshold selection across different data splits. The fold-wise optimal thresholds showed noticeable variability (0.15–0.50), indicating that the preferred operating point may shift depending on data partitioning and class composition. Nevertheless, the OOF-derived threshold of 0.49 remained within the upper portion of this observed range and was retained as the primary operating threshold because it was selected using the full cross-validation framework rather than tuned on the validation set. Furthermore, applying the optimized threshold to a held-out validation cohort provided an additional internal assessment of model behavior within the current dataset, supporting the stability of the proposed screening strategy despite the limited sample size.

Precision–Recall analysis revealed a typical class-imbalance pattern in which precision decreases sharply as recall increases [46], which is expected given the limited number of impaired samples. Nonetheless, an AP of 0.651 indicates that the model exhibits a stable ability to distinguish at-risk individuals beyond random classification. This property supports the suitability of the proposed model not as a stand-alone diagnostic tool, but as a first-line screening approach that can trigger additional clinical assessments or referral to confirmatory testing.

In the modality-wise performance comparison, the MMSE-only model achieved the highest AUC, reaffirming the strong diagnostic value of MMSE in current clinical practice. In contrast, sleep-only and activity-only lifelog models showed relatively lower performance; however, in multimodal models combining MMSE with lifelog features, balanced accuracy remained relatively comparable across several multimodal configurations. Notably, sleep lifelog features may provide exploratory complementary information for individuals whose MMSE scores fall near the decision boundary, although this remains exploratory. Conversely, the model that indiscriminately integrated all modalities did not yield clear performance gains, which may be attributable to information redundancy and noise effects associated with increased feature dimensionality. This observation also suggests that, in the current dataset structure, improving model architecture alone may not necessarily yield substantial performance gains unless larger-scale longitudinal data and raw temporal signal representations are available. Therefore, future studies may benefit from exploring sequence-aware deep learning approaches, such as Transformer-based temporal models, only when sufficiently large and temporally rich datasets can support such architectures.

Feature importance and stability analyses showed that variability-related indicators of activity and sleep patterns were repeatedly selected across multiple cross-validation folds. This finding is consistent with a growing body of research suggesting that intraindividual behavioral variability and dynamic instability may reflect early functional changes associated with cognitive decline and neurodegenerative processes [25,47]. These findings may be positioned within the emerging digital biomarker literature that emphasizes behavioral variability and dynamic instability as early indicators of functional decline in neurodegenerative conditions. Previous studies have reported that increased day-to-day variability in behavioral and physiological signals may capture subtle disruptions that are not fully reflected in mean-level measures alone [25]. In this context, the present findings may be interpreted as exploratory evidence that variability-oriented lifelog features could provide complementary information beyond traditional mean-level indicators used in cognitive assessment. However, this distinction was not directly tested in the current study and should be further investigated in future work. Overall, these findings suggest that temporal variability, rather than absolute levels of lifelog signals alone, may represent a potentially informative behavioral characteristic associated with cognitive impairment. However, this interpretation remains exploratory and requires further validation.

Key limitations include the small sample size of the validation set, uncertainty arising from inter-individual differences in lifelog data collection patterns, and the fact that the model was evaluated using a single dataset without independent external validation, which may limit the generalizability of the findings. In particular, because the present study was conducted using a single-center public dataset with a relatively limited number of cognitively impaired participants, caution is warranted when interpreting the robustness and broader clinical applicability of the proposed framework. Future studies should include larger multi-center cohorts, prospective longitudinal follow-up, and external validation across heterogeneous populations to determine whether the threshold stability, feature importance patterns, and screening utility observed in this study can be generalized to real-world clinical settings. Despite these limitations, this work demonstrates that threshold optimization and variability-focused feature interpretation can contribute to the development of lifelog-based multimodal screening models from a clinical screening perspective and may provide useful insights for designing data-driven early screening strategies in future digital health settings.

## 5. Conclusions

In this study, from a clinical screening perspective, we developed a multimodal binary classification model integrating MMSE with sleep and activity lifelog data and proposed a predictive framework to distinguish cognitively normal (CN) individuals from those with cognitive impairment (Impaired) via a cross-validation–based threshold optimization strategy. In particular, rather than adopting a fixed threshold (0.5), we derived an operational threshold that maximized balanced accuracy using out-of-fold (OOF) predictions, demonstrating that the clinically important sensitivity–specificity trade-off can be adjusted more rationally. Additional cost-sensitive analysis also suggested that the preferred operating threshold may vary depending on the relative clinical priority assigned to minimizing false negatives versus limiting unnecessary over-screening.

In validation analyses applying the optimal threshold (0.49), the model achieved an overall accuracy of 0.818, a balanced accuracy of 0.728, and a sensitivity of 0.571 for the impaired group. These results indicate that, even under limited sample size and class-imbalance conditions, the model exhibited classification behavior that limited missed impaired cases at a certain extent while relatively suppressing misclassification of the normal group. ROC and Precision–Recall analyses further supported that the proposed model is more suitable as an auxiliary tool for early-stage screening rather than for confirmatory diagnosis.

In the modality-wise comparison, the MMSE-only model showed the highest classification performance; however, the multimodal models combining sleep and activity lifelog features maintained a comparable level of balanced accuracy, and sleep lifelog features in particular showed potential to complement MMSE information. By contrast, indiscriminately integrating all lifelog modalities yielded limited performance gains, suggesting potential information redundancy and noise effects associated with increased feature dimensionality. These findings emphasize the importance of selective and purpose-driven utilization of lifelog data.

Furthermore, feature importance and stability analyses confirmed that, alongside MMSE item scores, variability-related lifelog indicators were repeatedly selected across multiple cross-validation folds. This suggests that cognitive impairment may be associated not only with shifts in mean levels but also with increased variability and instability in daily behaviors and physiological signals, and it highlights the potential for specific lifelog measures to be explored as digital biomarker candidates.

Overall, this study presents a threshold-based decision-making framework that considers interpretability and operational stability in clinical screening settings and exploratorily demonstrates the feasibility of using multimodal lifelog data as complementary information. The proposed framework may serve as a basis for future integration into home-based monitoring systems as a digital triage support tool that encourages timely clinical follow-up when abnormal behavioral patterns are detected in daily life. To realize this potential, future work should secure larger and more diverse multi-center cohorts, prospective longitudinal follow-up data, and independent external validation datasets to further verify threshold stability, model robustness, and the real world clinical utility of the proposed digital biomarkers.

## Figures and Tables

**Table 1:** Demographic and MMSE characteristics of the study population.

Group	n	MMSE Total Score (Mean ± SD)	Sleep Features (n)	Activity Features (n)
CN	111	27.70 ± 1.78	108	96
MCI	51	25.82 ± 3.28	108	96
Dementia	12	16.58 ± 8.03	108	96

**Table 2:** Cost-sensitive threshold analysis under different false-negative weighting scenarios on the validation threshold table.

Scenario	Preferred Threshold	Sensitivity	Specificity	Balanced Accuracy
Equal cost (FP = 1, FN = 1)	0.54	0.571	0.885	0.728
FN cost × 2	0.54	0.571	0.885	0.728
FN cost × 3	0.16	0.714	0.577	0.646
FN cost × 5	0.16	0.714	0.577	0.646

**Table 3:** Performance Comparison of Classification Models Across Input Modalities for CN vs. Impaired Screening.

Model/Modality	Input Modalities	No. of Features	AUC (Mean ± SD)	Balanced Accuracy (Mean)
MMSE only	MMSE	34	0.861 ± 0.042	0.731
Sleep only	Sleep lifelog	108	0.571 ± 0.061	0.552
Activity only	Activity lifelog	96	0.612 ± 0.058	0.579
MMSE + Sleep	MMSE, Sleep	142	0.769 ± 0.049	0.724
MMSE + Activity	MMSE, Activity	130	0.742 ± 0.053	0.701
Sleep + Activity	Sleep, Activity	204	0.641 ± 0.064	0.598
MMSE + Sleep + Activity	All modalities	238	0.698 ± 0.061	0.692

## Data Availability

The dataset used in this study is publicly available from AI Hub. The dataset “Dementia High-Risk Group Wearable Lifelog” is available at: https://www.aihub.or.kr/aihubdata/data/view.do?currMenu=115&topMenu=100&searchKeyword=%EC%B9%98%EB%A7%A4%20%EA%B3%A0%EC%9C%84%ED%97%98%EA%B5%B0%20%EC%9B%A8%EC%96%B4%EB%9F%AC%EB%B8%94%20%EB%9D%BC%EC%9D%B4%ED%94%84%EB%A1%9C%EA%B7%B8&aihubDataSe=data&dataSetSn=226 (accessed on 20 November 2025). Access may require registration and approval according to AI Hub policy, and use of the data is subject to AI Hub’s terms and conditions.
